# Postpartum Depression in Fathers: A Systematic Review

**DOI:** 10.3390/jcm13102949

**Published:** 2024-05-16

**Authors:** Pablo Álvarez-García, Rubén García-Fernández, Cristian Martín-Vázquez, Natalia Calvo-Ayuso, Enedina Quiroga-Sánchez

**Affiliations:** 1 Principality of Asturias Health Service, 33001 Oviedo, Spain; pablobarreo19@gmail.com; 2 SALBIS Research Group, Department of Nursing and Physiotherapy, Faculty of Health Sciences, Campus de Ponferrada, Universidad de León, 24401 León, Spain; ncala@unileon.es (N.C.-A.); equis@unileon.es (E.Q.-S.); 3 Nursing Research, Innovation and Development Centre of Lisbon (CIDNUR), Nursing School of Lisbon, 1600-190 Lisbon, Portugal; 4 Department of Nursing and Physiotherapy, Campus de Ponferrada, Universidad de León, 24401 León, Spain; cmartv@unileon.es

**Keywords:** depression, postpartum, postpartum period, father

## Abstract

**Background/Objectives:** Postpartum depression is usually defined as a major depressive episode that occurs shortly after childbirth. This condition is most commonly found in females; however, paternal postpartum depression has begun to attract more research attention. This study aims to identify different instruments for measuring this mental health problem and to detect risk factors as well as the main sources of resilience in paternal postpartum depression. **Methods:** A literature review was conducted following the PRISMA method. **Results:** After analyzing 10 articles, it was determined that the Edinburgh Postpartum Depression Scale is the most widely used instrument for the diagnosis of postpartum depression in the female population, and after several studies, it has already been validated for the male sex. After several studies were analyzed to highlight the main risk factors for paternal postpartum depression, it was established that the most influential factor is male gender role stress. These findings highlight the traditional role of fathers today. Most health professionals see the mother as the priority. **Conclusions:** Paternal depression is a major problem for mothers and fathers today, as well as for the newborn. As time goes on, there is a growing need to incorporate fathers into current and future mental health programs to be able to provide the necessary support.

## 1. Introduction

According to data reported by the World Health Organization (WHO), postpartum depression is projected to become the second leading cause of disability and the most common mental illness worldwide. Presently, it affects 300 million individuals, exhibiting a higher prevalence in women than in men [1]. The WHO also indicates that between 10% and 20% of women may experience depression at some point after childbirth, which equates to approximately one in ten women. It is noteworthy that this condition can affect individuals of any age, race, educational level, or socio-economic status [2,3]. Nevertheless, multiple studies underscore that between 20% and 40% of women in developing countries experience postpartum depression, primarily due to inadequate access to healthcare services [4,5,6]. Furthermore, the prevalence of depression can vary significantly by geographical location. Research demonstrates that the global prevalence rate of depression can range from 1% to 39% depending on the region. Studies conducted in Latin America found a prevalence rate of 33% overall, with studies conducted in Brazil and Mexico reporting a prevalence of 26.3% and 23.8%, respectively. From a European perspective, the prevalence also fluctuates depending on the location. Studies conducted in Norway estimate a rate of 9%, compared to Turkey, which has a rate of 21.2% [4,5].

In Spain, the prevalence rate is 25.8%. The United States exhibits a comparable value, yet due to its vast size, the prevalence rate ranges between 8% and 26% [7]. In Africa, studies conducted in South Africa found a prevalence of 34.7%, while those in Ethiopia reported a prevalence of 11.8% [4].

Historically, postpartum depression has been primarily associated with women. However, in recent years, there has been increasing recognition of the impact that this condition can have on fathers [6]. Postpartum depression, regardless of gender, is considered a form of depression that can range from moderate to severe. Given the potential complications associated with depression, it is increasingly important to acknowledge its symptoms, prevalence, associated challenges, and treatment options in order to provide appropriate support [7]. While men do not undergo the same physiological, anatomical, and hormonal changes as women during childbirth, they experience significant psychological changes, particularly related to their new role and its responsibilities [6].

A meta-analysis involving over one million participants across more than 30 countries has established that postpartum depression can also affect men, with prevalence rates ranging from 9.76% during the prenatal period to 8.75% in the postnatal period, which can persist for over a year. A study conducted in Chile, which analyzed a male population using the Edinburgh Postnatal Depression Scale, reported a prevalence of 18.5% [7]. However, the overall prevalence of postpartum depression varies geographically. A literature review revealed prevalence rates ranging from 1.8% to 47% in Turkey and Sweden, respectively [7].

In recent years, fathers have taken on a more active role during childbirth. Their involvement is associated with better pregnancy outcomes, improved maternal well-being, and even the mental, physical, emotional, and social development of the baby. However, several studies have highlighted the lack of support received by fathers during their transition to fatherhood, particularly from healthcare professionals. For instance, some fathers, following the loss of a baby, felt that their role was merely to support the mother [4,6]. Current studies emphasize the barriers preventing men from seeking help, especially in a society where men often conceal their vulnerabilities and strive to appear strong. Additionally, healthcare professionals may inadvertently contribute to this barrier by making fathers feel excluded. As previously discussed, the etiology of postpartum depression is multifactorial, with various risk factors such as sleep disturbances, premature birth, or depression in the partner [6,8]. Postpartum depression in fathers presents a significant challenge for public health, not only due to its impact on paternal well-being but also because of its potential effects on the mother and newborn. It is imperative to raise awareness among fathers about the existence of support networks to mitigate the occurrence of these mental disorders. For these reasons and more, it is essential to incorporate fathers into current and future perinatal mental health programs [5].

Given the limited understanding of the global perspective on postpartum depression in fathers, this article aims to identify different instruments for measuring this mental health problem and to detect the risk factors as well as the main sources of resilience in paternal postpartum depression.

## 2. Materials and Methods

A systematic review of the literature was conducted. This review was based on the Preferred Reporting Items for Systematic Reviews and Meta-Analyses (PRISMA) methodology [9]. A protocol was registered with the Open Software Foundation (OSF) (https://osf.io/r32mf (accesed on 19 March 2024)).

The SPIDER (Sample, Phenomenon of Interest, Design, Evaluation, and Research) [10] tool was used to determine the following research question: What are the most effective instruments for measuring paternal postpartum depression, associated risk factors, and sources of resilience, and what studies have addressed these issues (Table 1)?

A search of the Web of Science, Scopus, MEDLINE, and CINAHL databases was carried out during October–November 2023. The keywords used for the search were “pregnan*”, “father”, and “depression” using the Boolean operator “AND”. Only articles published between 2018 and 2023 that were open access and published in Spanish or English were included. In addition, articles whose titles associated postpartum depression with another type of pathology, such as COVID-19, were excluded. These criteria and filters were used for all the databases used in our review.

Assessment of methodological quality: The methodological quality of studies eligible for inclusion in the systematic review were assessed using the risk-of-bias checklist (Table 2). If necessary, the authors of the articles were contacted for clarification or additional information. Any disagreements between the reviewers were resolved through recourse to the third reviewer. Data transformation: Quantitative data were converted into qualitative information to facilitate integration with data extracted from qualitative studies through narrative description of the quantitative data. Data synthesis and integration: The review followed an integrated convergent approach according to the JBI methodology [11]. The extracted data were categorized and integrated according to the findings.

## 3. Results

**Figure 1:**
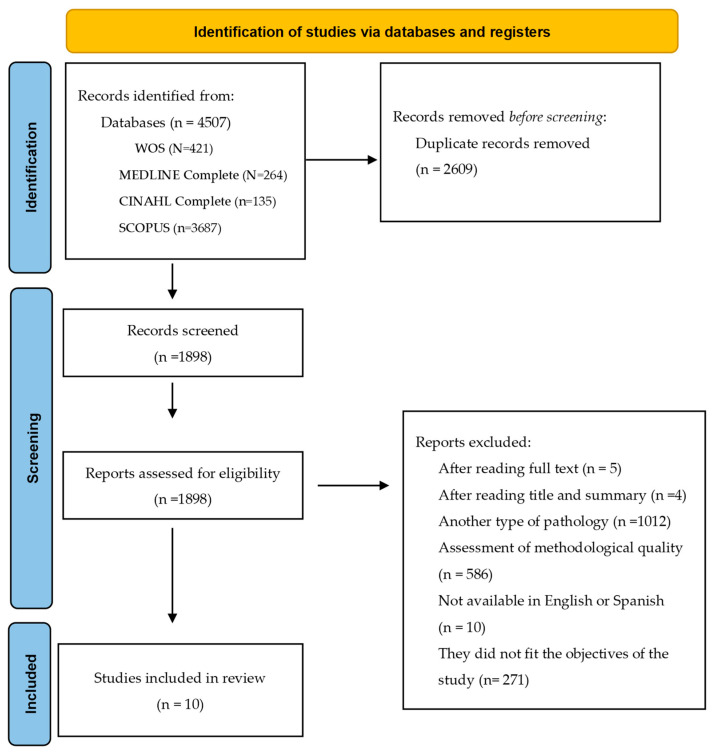
PRISMA flow diagram.

A total of 4507 articles were retrieved from the search. The article selection process is described in Figure 1, which shows the PRISMA diagram. Finally, the systematic review included a total of 10 studies.

After a thorough analysis of the articles, we used a thematic approach to evaluate the data according to our objectives, obtaining the results described in Table 3.

## 4. Discussion

According to the study conducted by Rigmor R.B. [13], there has been an increase in research focusing on and measuring paternal postpartum depression (PPD), indicating growing interest in paternal depression concerning fatherhood. After analyzing 59 studies, we identified a total of 13 instruments. However, only four instruments have been validated. Among these, the EPDS (Edinburgh Postnatal Depression Scale) is considered the most valid currently available tool for PPD diagnosis. Apart from this instrument, only one other is specifically related to the postpartum period and pregnancy: the PDSS (Postpartum Depression Screening Scale). Additionally, only two other instruments are specifically designed to analyze symptoms in males: the PAPA (Paternity Adjustment and Paternal Attitudes Questionnaire) and the GMDS (Gotland Male Depression Scale). None of the instruments were created solely for PPD diagnosis. The two instruments related to the postpartum period, the EPDS and PDSS, were primarily designed for the female population. The EPDS is currently the most widely used instrument for assessing postpartum depression in women. With a total of eight validation studies, it can now also be used as a diagnostic tool for postpartum depression in fathers. The study conducted by Kothari et al. [12] supports the previously mentioned study and views the EPDS as the most appropriate instrument for diagnosis. The EPDS consists of a 10-item self-report questionnaire, with each item having four possible responses ranging from 0 to 3 based on severity, with a maximum total score of 30; a score of 10 or higher indicates probable postpartum depression but not its severity. Additional research is required to ascertain whether there is a necessity for the development of a more precise instrument.

Regarding the geographical distribution of the findings, studies conducted in Australia [12,19], Pakistan [14], China [16], Italy [17], New Zealand [18], and Great Britain [20], among others, were identified, showcasing a significant heterogeneity of study locations, as well as a dearth of research in Europe and America.

In this context, concerning the study samples, it is evident that both individual male parents [12,14,16,17,18,19,20] and couples [15,21] have been investigated. Notably, some studies specifically reference heterosexual couples [18], thus overlooking new realities of non-cis heteronormative couples, which represents a compelling avenue for further research. As for the most pertinent sociodemographic data, a distinct profile emerges with the majority of research indicating an average age exceeding 30 years [12,14,17,18], predominantly white or Caucasian participants [12,15,18,20] with over 50% having previous children [12,14] and being employed [12,14,15,16,18,20]. Employment may serve as a protective factor [16], yet it can also pose concerns as the pressure to provide economic stability through work can become a stressor in the context of parenthood. Further investigations exploring the interplay of factors such as employment are warranted.

Moreover, substantial heterogeneity was observed across the studies in two key aspects: participant recruitment strategies and criteria for inclusion and exclusion pertaining to mental health histories and serious neonatal illnesses. Participant recruitment employed various methods, with medical centers and hospitals being the most prevalent [12,14,15,16,17,21], alongside recruitment via social networks and leaflets [18,19]. Notably, two studies employed markedly different participant selection approaches despite both being conducted within healthcare settings. Specifically, Atif et al. recruited participants through mothers, citing cultural reasons [14], while Agostini et al. recruited from the intensive care unit (ICU) [17]. Furthermore, diversity was evident in criteria related to mental health backgrounds and neonatal illnesses across the studies, with some including individuals with prior mental health histories and stressful life events [15,19], and others excluding participants with psychiatric backgrounds and serious neonatal illnesses [16,17]. Thus, the variability in participant selection methods and criteria may introduce confounding factors, warranting standardized approaches to facilitate robust analyses.

The design and methodology of the studies reviewed encompass a mix of qualitative [18,19,21], mixed-methods [12], and quantitative [14,15,16,17,20] research, thereby offering the objectivity of quantitative data and the exploratory nature of qualitative data. Notably, there is a dearth of clinical trials, which could offer higher-quality evidence and enhanced rigor.

Regarding risk factors, the study conducted by Chhabra et al. [20] proposes unplanned pregnancy, sleep disturbance, work–family conflict, MGRS (male gender role stress), marital distress, and maternal depression as significant contributors. The financial burden associated with having a baby often increases due to unforeseen expenses. An unplanned pregnancy, catching parents off guard, can induce anxiety and financial strain, especially among low socio-economic groups and/or the unemployed. Sleep disturbance, often resulting in fatigue and irritability, can hinder father–child interaction and potentially lead to developmental issues in the baby’s cognitive and behavioral domains. Work–family conflict, crucial for maintaining a quality life, may result in depressive symptom development if a balance between work and family responsibilities cannot be achieved [20].

Regarding male gender role stress, traditional societal norms often dictate the expectation of being the ‘family breadwinner’ who is economically responsible for the family. Failure to meet these social expectations can lead to stress, potentially resulting in depression. Another risk factor is marital distress within the relationship. The arrival of a new baby may reduce the time parents spend together or hinder their ability to communicate about their challenges. This lack of communication and emotional connection may contribute to psychological distress in parents. Lastly, maternal depression has been identified as a predictor of paternal depression. Given the interconnected nature of the couple’s environment, it is not uncommon for them to share stressful factors and experience depressive symptoms together [15,20].

The study conducted by Atif et al. [14] provides clear evidence of certain risk factors, such as sleep disturbance, maternal depression, and male gender role stress. According to these authors, shared interpersonal and environmental stressors can impact both members of a couple, contributing to the development of depressive symptoms. It has also been reported that sleep disturbance is highly associated with paternal depression, particularly among first-time fathers. Lastly, regarding male gender role stress, societal norms often delineate traditional gender roles based on outdated values. Men are frequently expected to embody characteristics of physical strength and assertiveness, along with responsibilities such as providing for their families, and displaying vulnerability may be stigmatized [14].

There is a relationship between the risk factors identified in the studies conducted by Johansson et al. and Chhabra et al., both of which mention work–family conflict. In these studies, parents were actively involved in their children’s lives; however, they expressed feelings of inadequacy due to the challenge of balancing work and personal responsibilities [20,21].

Cui et al., compared with the study conducted by Chhabra et al., also highlight marital distress as a risk factor, among others. An increase in conflicts in the marital relationship has been associated with a fivefold greater likelihood of postpartum paternal depressive symptoms [16,20]. In addition, these findings emphasize predisposing factors such as vulnerability and employment status, which are predominantly attributed to the socio-economic adversities concurrent with childbirth.

The most prevalent risk factor identified appears to be male gender role stress. The majority of men in these studies perceived themselves primarily as providers of support for their partners and children, with their main role being the “family breadwinner”. Many fathers reported that midwives, family members, and friends also reinforced this perception of their primary role. These findings underscore the traditional supportive role that fathers commonly assume in contemporary society [14,18,20].

It is also worth noting the studies conducted by Agostini et al. [17], aimed at determining the characteristics of paternal postpartum depression (PPD) during the first postpartum year and exploring the potential influence of prematurity severity as a risk factor. The researchers found no significant differences in symptomatology between the fathers of premature babies and those of full-term babies. However, a discrepancy was observed in depressive symptomatology over time. The Edinburgh Postnatal Depression Scale (EPDS) served as a diagnostic instrument, revealing high scores at 3 months postpartum, followed by a decline at 6 months, and a more notable decrease at 9 months. These findings imply that the likelihood of paternal depression reaches its highest point in the early postpartum period, presenting an emotional and psychological hurdle, given that the newborn’s needs are most prominent in the early stages.

According to the study conducted by Kumar et al. [15], as a rule, women tend to receive support from a wide variety of interpersonal relationships and rely more on external signals to regulate their emotions. From the context of pregnancy, women may benefit from not being alone but rather being part of a much larger community facing pregnancy-related issues. Conversely, men tend to pay more attention to internal signals when making judgments about their emotions; these findings allow us to determine that men find a source of resilience in a satisfying intimate partner relationship.

Ghaleiha et al. [18] characterize a fulfilling couple relationship as the primary source of support for men; however, during these periods, mothers may not always be able to fulfil that supportive role. The findings indicate that family and friends serve as crucial sources of practical and emotional support. Additionally, this study, in conjunction with Johansson et al. [21], reveals the challenges fathers encounter when seeking help. Most fathers normalized their symptoms and did not perceive their situation as “serious” enough to reach out to support networks. This phenomenon may have arisen from the expectation among these fathers that experiencing such levels of stress is typical, or perhaps they were not adequately informed about the potential health risks associated with it. Therefore, it is crucial to educate parents about the symptoms involved [18,21].

Finally, most fathers perceived that healthcare professionals prioritize women. Fathers expressed that their main barrier to addressing problems was social stigma, in which they are seen as the main source of support for both the woman and the baby. Several fathers suggested that healthcare professionals only considered the mother’s well-being. This lack of recognition by healthcare professionals further reduced fathers’ confidence in seeking help. These data conclude that paternal mental health has a noticeable effect on the father–child relationship, as well as on their physical and cognitive development. This indicates the need to identify both maternal and paternal well-being [19].

The evidence presented in this review uncovers several new avenues for research. Firstly, there is an imperative to develop a validated tool specifically tailored for assessing postpartum depression in fathers, thereby ensuring the provision of more robust and high-quality evidence. Furthermore, there is a pressing need for studies that explore emotional dynamics within non-cisgender heteronormative families and among parents. Methodologically, it is crucial to design randomized clinical trials employing mixed methods to enhance the rigor of existing evidence. Additionally, the standardization of study conditions is indispensable for enabling more objective comparisons.

## 5. Conclusions

After analyzing various studies, it has been determined that depression constitutes a comprehensive mourning process for parents during both the pregnancy and postpartum periods. Our study identified a total of 13 instruments for diagnosing paternal depression; however, it was concluded that the most reliable scale for diagnosis, given its characteristics, was the EPDS (Edinburgh Postnatal Depression Scale). An analysis of the main risk factors was also conducted, revealing that males share several risk factors with females, making it relatively straightforward to identify some of them, such as unwanted pregnancy, work–family conflict, and marital distress, among others. However, the most influential factor in the development of potential postpartum paternal depression was MGRS (Male Gender Role Stress), a factor exclusively influential in fathers. This underscores the importance of considering traditional gender stereotypes when evaluating a father, as men adhering to such stereotypes are more susceptible to experiencing postpartum depression.

Finally, we sought to examine the primary sources of resilience among parents, determining that the partner emerged as their greatest source of support. Health professionals were noted as a deficient source of support, often prioritizing the mother as the primary focus. Paternal postpartum depression constitutes a challenge for fathers themselves, mothers, and the newborn. The inclusion of fathers in current and future mental health programs is increasingly imperative to raise awareness about this form of depression and to provide the necessary support to new parents in their transition to parenthood.

## Figures and Tables

**Table 1:** SPIDER strategy.

Sample	Men with Paternal Postpartum Depression and/or Their Partners.
Phenomenon of Interest	What are the different instruments used to measure paternal postpartum depression and its risk factors, as well as the main sources of resilience?
Design	Studies addressing the detection and measurement of paternal postpartum depression.
Evaluation	To identify the most effective instruments, relevant risk factors and sources of resilience in paternal postpartum depression.
Research type	Quantitative and qualitative.

**Table 2:** Assessment of methodological quality.

Study	D1	D2	D3	D4	D5	Overall
A. Kothari et al. (2022) [12]	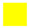	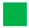	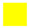	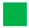	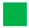	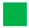
R. Berg et al. (2022) [13]	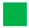	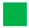	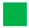	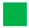	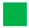	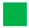
M. Atif et al. (2022) [14]	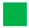	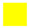	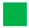	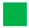	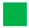	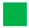
A. Kumar et al. (2022) [15]	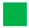	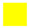	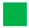	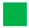	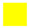	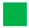
Y. Cui et al. (2021 [16])	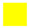	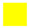	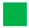	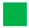	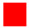	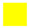
F. Agostini et al. (2022) [17]	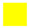	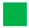	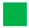	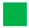	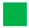	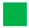
A Ghaleiha et al. (2022) [18]	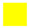	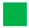	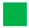	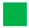	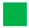	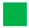
S. Hambidge et al. (2021) [19]	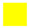	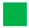	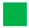	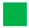	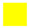	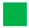
J. Chhabra et al. (2022) [20]	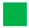	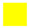	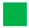	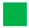	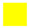	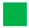
M. Johansson et al. (2020) [21]	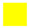	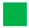	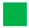	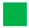	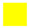	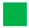

D1: Randomization process; D2: Deviations from intended intervention; D3: Missing outcome data; D4: Measurement of the outcome; D5: Selection of the reported result; ■ Low risk; ■ Some concerns; ■ High risk.

**Table 3:** Results of the systematic review.

Author and Year	Type of Study	Aim of the Study	Study Population	Relevant Results
A. Kothari et al. (2022) [12]	Prospective mixed methods studies	To explore the prevalence of symptoms of depression and traumatic stress in parents in the context of poor fetal, neonatal, and maternal outcomes.	28 fathers whose partners had experienced a pregnancy or complicated childbirth	The obtained results were measured using two scales, EPDS and IES-R, at two different time points. Scores were generally high. This study provides evidence for parental distress/depression after birth complications and highlights the need to score and intervene for mental health conditions in parents during and after pregnancy.
R. Berg et al. (2022) [13]	Scoping review	To identify instruments used to measure depressive symptoms among parents during pregnancy and the puerperium and describe instrument characteristics and measurement properties.	Not applicable	An evaluation of 12 validation instruments was conducted to determine their credibility and reliability. None of these provided information on responsiveness. The EPDS was found to be the most widely studied and most correct instrument.
M. Atif et al. (2022) [14]	Descriptive cross-sectional observational study	To identify risk factors for paternal postpartum depression in Pakistani men and incorporate them into mental health programs.	73 men contacted via their wives	During this study, almost a quarter of all participants tested positive for paternal postpartum depression. Maternal depression and sleep disorders were the most influential risk factors.
A. Kumar et al. (2022) [15]	Correlation study	To identify and promote sources of resilience to past stressful situations in parents to prevent postpartum depression.	159 couples (159 women and 159 men).	The study establishes a relationship between past stressful situations and the severity of depressive symptoms. To reduce this relationship, self-compassion, i.e., kindness or common humanity as a source of resilience, was promoted in mothers. In fathers, self-compassion was also promoted, but this time in the form of mindfulness, in addition to which the fathers had an extra source of resilience, i.e., the satisfaction of the intimate relationship.
Y. Cui et al. (2021) [16]	Cross-sectional study	To examine the incidence of and factors related to paternal postnatal depression in Guangzhou, China.	190 fathers	There was a high prevalence of postnatal depressive symptoms in men for 6 months from the birth of the baby; the most striking risk factors were vulnerable personality and unemployment. The need to periodically evaluate depressive symptoms in parents with a more extensive evaluation system is evident.
F. Agostini et al. (2022) [17]	Longitudinal research	To explore levels of paternal postpartum depression at 3, 9, and 12 months postpartum, including the possible influence of preterm birth, low birth weight, and partner depression.	153 fathers of 33 extremely-low-birth-weight infants, 42 very-low-birth-weight infants, and 78 full-term infants, respectively	The results emphasize that the severity of paternal postpartum depression is not influenced by prematurity or low birth weight of the baby, nor is it influenced by a partner’s depression. These findings are justified by the male inability to express feelings of fear that are culturally and socially unacceptable.
A Ghaleiha et al. (2022) [18]	Qualitative research	To understand the attitudes of fathers in the process of becoming fathers, how they coped with this change, and where they sought advice and support.	11 fathers	Fathers in their transition to fatherhood see themselves as a figure of stability for their wives, which they see as a challenge. This study emphasizes the need to develop new forms of support for future fathers.
S. Hambidge et al. (2021) [19]	Qualitative research	To gain an understanding of the mental state of parents during the pre- and postnatal period.	29 fathers	A high number of parents experienced mental health problems during the prenatal and postnatal period. This study highlights the limited influence of health professionals in improving or supporting parents, who are relegated to second place.
J. Chhabra et al. (2022) [20]	Transversal mixed methods studies	To qualitatively identify possible risk factors for the development of paternal perinatal anxiety and depression and to quantitatively determine which factors predispose fathers to these pathologies.	Qualitative study, n = 13; quantitative study, n = 252; men 18 years of age and older, with a pregnant partner and/or an infant under the age of 12 months, who spoke and comprehended basic English and were currently residing in Australia	This mixed study shows that the main risk factors for paternal perinatal depression and anxiety are maternal depression, male gender role stress, marital distress, and work–family conflict. Sleep disturbance stands out as it has not been studied before, and unwanted pregnancy was identified as a risk factor only in the qualitative study.
M. Johansson et al. (2020) [21]	Qualitative research	To conduct a qualitative analysis of parents’ experiences of postpartum stress and depression through individualized interviews using interpretative phenomenological analysis.	10 mothers and 5 fathers	The obtained qualitative results highlight the severity with which depressive symptoms and/or stress can influence the development of daily life as well as a couple’s relationship. They emphasize the need for a more individualized healthcare approach for both parents in their transition to parenthood.
